# Experiences with and perception of patient-reported outcome measurement in patients undergoing knee and hip replacement in Germany

**DOI:** 10.1186/s41687-023-00618-3

**Published:** 2023-07-24

**Authors:** Adriana N. König, Sebastian Himmler, Peter Buschner, Leonie Sundmacher

**Affiliations:** 1 Technical University of Munich, Munich, Germany; 2 Krankenhaus Barmherzige Brüder München, Munich, Germany

## Abstract

**Background:**

Patient-reported outcome measures - PROMs - have been developed to provide an assessment of patients’ physical function, symptoms, and health-related quality of life. With patient-centered care becoming increasingly important, several national strategies have been initiated for PROM measurement. However, Germany is only at the beginning of this process. The objective of this study is to assess patients’ experience with and perception of completing PROMs in patients undergoing knee and hip replacement in Germany.

**Methods:**

This study used survey data from patients undergoing hip or knee replacement surgery in a hospital in Germany. Before surgery, patients completed a PROMs survey. After at least 6 months, patients were re-contacted to fill in a questionnaire about their experiences with and perception of the PROMs data collection.

**Results:**

Most patients either agreed or totally agreed that the time to fill in the questionnaire was appropriate (89%), that the purpose of the PROMs collection was clear (85%), that the questionnaire’s content applied to their appointment (73%), and that this systematic assessment was beneficial (81%). The corresponding proportions were 54% for feeling productive while waiting and 50% for feeling that the information in the questionnaire affected the patient-doctor interaction positively. Only few significant associations were found between patient characteristics and the favorability of patients’ ratings. There were no significant differences between hip and knee replacement surgery patients regarding the favorability rating on any survey question.

**Conclusions:**

The results of this study suggest that PROMs collection in the context of hip and knee replacement surgery is practicable and partly also perceived beneficial by patients. Orthopedic procedures could serve as a starting point for broader use and routine PROMs collection in Germany.

## Introduction

Patient-reported outcome measures (PROMs) have been defined by the Food and Drug Administration as “a measurement of any aspect of a patient’s health status that comes directly from the patient (i.e., without the interpretation of the patient’s responses by a physician or anyone else)” [1, para.2]. They have been developed to provide an assessment of patients’ physical function, symptoms, and health-related quality of life [2]. While national initiatives for PROMs collection, registries and standards exist in England, Sweden, Denmark or the Netherlands, Germany is only starting to gather first experiences with PROMs in the context of pilot- and research projects [3]. One disease area where PROMs collection is most widely implemented internationally is knee and hip replacement [4]. In Germany, little is known about outcomes from the patient perspective in these indications, especially outside of clinical studies, while hip and knee replacement are among the most common surgeries in Germany with a total of 405,548 procedures in 2021 [5]. This hampers both the assessment of the quality of different providers and the effectiveness of the use of innovative procedures [6].

While the use of PROMs in these indications is now advocated by recent guidelines [6, 7] in Germany, it is also expected that implementation of PROMs in routine care and quality assurance in Germany will be pushed by institutions like the Federal Joint Committee, the Institute for Quality and Efficiency in Health Care or the Institute for Quality Assurance and Transparency in Health Care (IQTIG) in the near future.

Given the growing importance of PROMs and that it is patients that who must complete them, it is relevant to assess how well PROMs measurement is received. If their purpose is not clear to patients, or the response burden is considered too high, the quality of the collected data could suffer. It has been shown that the use of PROMs can improve patient–provider communication and patient participation [8, 9], and patients generally perceived PROMs collection as acceptable [9–12]. At the same time, some of these results may be specific to the examined context and condition. Patient satisfaction with and experiences of PROMs collection in a clinical setting have not yet been examined in Germany. Thus, the objective of this study is to assess patients’ experience with and perception of completing PROMs in patients undergoing knee and hip replacement in one surgical center in Germany.

## Methods

The following paragraphs outline the conduct of the initial PROMs collection prior to the respective surgeries, the design and administration of the follow-up survey on the experiences with and perception of the initial PROMs collection, and the statistical analysis of the corresponding survey data.

### PROMs data collection

The PROMs data collection that this study refers to was conducted in conjunction with the MobilE-PRO project. This project included PROM measurement in patients with primary hip and knee replacement at a hospital in Munich [also reported on here: 13]. During the visit at the clinic and prior to their surgery, patients were asked to participate in the underlying study. The PROMs questionnaire was digitally administered via tablets at the initial assessment on-site and no explicit assistance for filling out the survey was provided. The assessed PROMs were the five-dimensional EQ-5D-5L, a generic health-related quality of life instrument [14], and the 24-item, disease-specific Western Ontario and McMaster Universities Osteoarthritis Index (WOMAC) [15]. The use of both measures in this context was also recommended by the German association of endoprosthetics [6]. The MobilE-PRO project was approved by the ethics committee of the Ludwig-Maximilians-Universität München (reference number: 18-274).

### Survey for assessing perception of PROMs collection

All patients who provided PROMs data before their surgery were re-contacted by the hospital, as long as the surgery had taken place at least 6 months previously. Patients answered the follow-up survey between January 2021 and February 2022. The survey was conducted in conjunction with a routine follow-up questionnaire of the hospital, which is scheduled to take place 6, 12 and 60 months after surgery. Comparable time frames have also been used in previous research on PROMs [16–18]. In the time frame between 08/2019 (start of the MobilE-PRO project) and 08/2021 (last patient response in February 2022 minus minimum of six months between surgery and follow-up), a total of 4490 patients were operated in the hospital. The questionnaire about the experience with the PROMs collection was sent to all patients providing their e-mail addresses and their consent to be contacted by the hospital. The survey was administered online. After a short description of the survey’s purpose, i.e., assessing patient satisfaction with the PROMs collection, patients were asked to provide informed consent for this follow-up survey.

To measure patients’ experiences with and perception of completing the EQ-5D-5L and WOMAC PROMs collection, which took part prior to their surgery, six questions were included in the follow-up survey. These questions were previously used by [10] in a similar study. The initial English versions and the German translation are shown in Table 1. The translators were fluent in both English and German and familiar with the study to ensure the accuracy of the translations. To validate the translations, back-translation was used to ensure that the meaning was accurately conveyed in the translated version. However, no pilot testing or cognitive interviews were conducted. The first two questions related to patients’ experiences with filling in the PROMs, while the remaining four questions were aimed at eliciting patients’ general perception of the PROMs collection. The possible responses to all questions were defined based on a five-point Likert scale from “totally disagree” to “totally agree”. Patients were explicitly given the opportunity to skip questions to prevent them from responding to aspects they might not recollect. At the end of the survey, patients could express their thoughts about the PROMs collection in an open text field.

**Table 1:** Survey questions about PROMs collection

Abbrevivation	English version	German translation
Time	The time to complete the questionnaires was appropriate	Die Zeit für das Ausfüllen der Fragebögen war angemessen
Purpose	The purpose of these questionnaires was clearly explained before completing them	Der Zweck dieser Fragebögen wurde vor dem Ausfüllen klar erklärt
Appointment	I feel/felt that the content of this questionnaire applied to my appointment	Ich habe/hatte das Gefühl, dass der Inhalt des Fragebogens mit meinen Termin zu tun hatte
Interaction	I feel/felt that the information in the questionnaires affected my interaction with my doctor in a positive way	Ich habe/hatte das Gefühl, dass die Informationen in den Fragebögen meine Interaktion mit meinem Arzt positiv beeinflusst haben
Involvement	I had a feeling of involvement and/or productivity during my wait	Ich hatte während meines Wartens ein Gefühl der Beteiligung und / oder Produktivität
System	Having a system that allows my doctors to more accurately assess how I am doing is a benefit of [name of hospital]	Ein System zu haben, mit dem meine Ärzte genauer einschätzen können, wie es mir geht, ist ein Vorteil der [Name des Krankenhauses]

### Participants and exclusion criteria

Overall, 252 patients completed the survey about the PROMs collection and provided informed consent. We excluded participants who stated in the open field that they did not understand the survey or patients who did not remember completing PROMs (N = 12), patients who did not indicate the date of their surgery (N = 1) or who received their surgery more than 18 months ago (N = 9). We assumed that adequate recollection was unlikely in these instances.

### Statistical analysis

The statistical analysis was performed in R [19]. First, the frequencies of the response categories to the six questions about the PROMs collection were calculated and plotted. Second, ordinal logistic regressions were estimated separately for each survey question to detect associations between patient characteristics and survey responses [cf. 20]. The proportional odds assumption was violated for two independent variables in the model on application to appointment, but results from a partial proportional odds model were comparable. Thus, for simplicity and better comparability, a proportional odds model was reported. Coefficients were exponentiated to obtain odds ratios.

The patient characteristics included in the models were gender, age, place of residence, length of the time period since surgery, and type of replacement surgery. All variables except age were coded as binary variables (see notes in Table 3). Age was measured in seven age groups, relating to varying age brackets, which were necessary to ensure anonymity of respondents (see Table 2 for categorization). An interaction term between age and gender was included in all models. Significant interactions were visualized with the “sjPlot” package [21].

To examine whether the PROMs collection was perceived differently between hip and knee replacement patients, Chi-square tests were used to compare responses between these two groups. To this end, we dichotomized survey responses to the six Likert-scaled questions into “favorable” (“agree” and “totally agree”) and “unfavorable” (“indifferent”, “disagree”, “totally disagree”) [see 10, for a similar categorization].

## Results

The number of patients included in the analysis was 230. The characteristics of the sample are summarized in Table 2. The sample was balanced regarding gender, and the majority were 70 years or older. Most patients received hip surgery (75.2%), with the time since surgery being between 6 and 11 months for 59.1% of the sample.

**Table 2:** Characteristics of survey respondents

Gender (n (%))	
Female	114 (49.6)
Male	116 (50.4)
Age groups in years (n (%))	
Below 50	8 (3.5)
50 to 59	28 (12.2)
60 to 64	29 (12.6)
65 to 69	41 (17.8)
70 to 74	48 (20.9)
75 to 79	42 (18.3)
80 to 89	34 (14.8)
Place of residence (n (%))	
Munich	104 (45.2)
Outside Munich	126 (54.8)
Type of replacement surgery (n (%))	
Hip	173 (75.2)
Knee	57 (24.8)
Time period in months (n (%))	
12 to 17	94 (40.9)
6 to 11	136 (59.1)
Observations	230

**Fig. 1:**
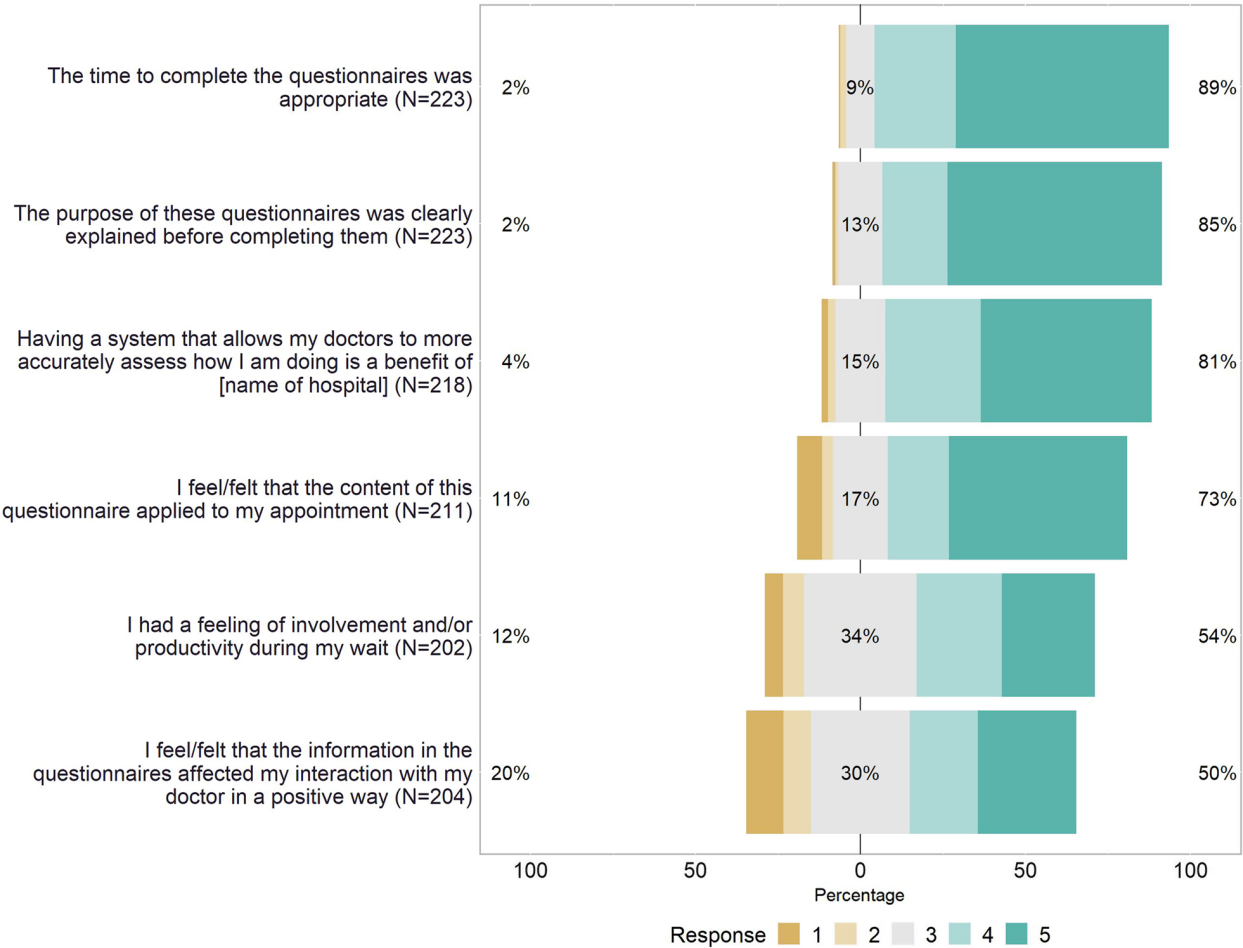
Frequencies of response categories as a percentage from 1 = “totally disagree” to 5 = “totally agree”. Note: Not all patients responded to every question

Eighty-nine percent of patients agreed or totally agreed with the statement that the time to complete the questionnaire was appropriate (Fig. 1). This number was 85% for the statement on a clear explanation of the questionnaires’ purpose. The majority also felt that the content of the questionnaire applied to their appointment (73%) and evaluated the systematic assessment of their condition as beneficial (81%). Around half of patients had a feeling of involvement or productivity when filling in the PROMs during their wait (54%) or felt that the questionnaires’ information positively affected the interaction with their doctor (50%).

Table 3 presents the results of the proportional odds models where coefficients are represented and interpreted as odds ratios. They indicate the associations between being female, living in Munich, having had hip replacement surgery and time since surgery of 12–17 months and the questions on experiences with and perception of PROMs collection. In addition, the coefficient for age describes the association between increases in the age groups and the questions on experiences with and perception of PROMs collection. Significant associations between patient characteristics and the perception of the PROMs collection were found in three survey questions. Living in Munich was associated with a more favorable feeling that the questionnaire purpose was clearly explained (OR = 2.019). Being female (OR = 0.207 and OR = 0.315 respectively) and the interaction between being female and age (OR = 1.396 and OR = 1.287 respectively) were significantly associated with the questions on positive interaction and feeling of involvement.

**Fig. 2:**
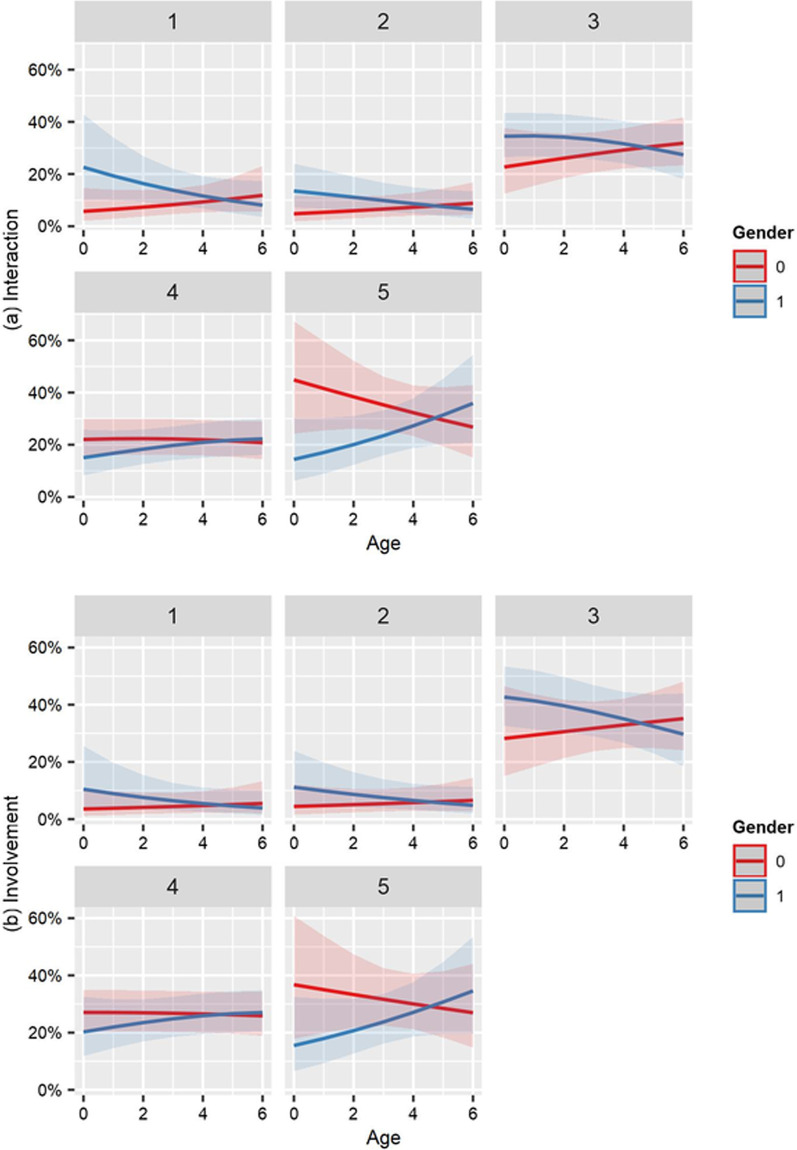
Predicted probabilities for survey questions on positive interaction and feeling of involvement. Numbers on top represent response levels (1 = “totally disagree”). Gender dummy 1 equals female. Age groups categorically coded with 0 being < 50 to 6 being 80–89

Figure 2 illustrates the interaction effects for these two questions. The plots suggested lower predicted probabilities for response level five (“totally agree”) in females, except in the highest age group, but gender differences were not statistically significant.

**Table 3:** Proportional odds models by patient-reported rating

Question	Time	Purpose	Interaction	Involvement	System	Appointment
Female	0.729	0.370	0.207**	0.315*	0.629	0.838
	[0.633]	[0.657]	[0.565]	[0.587]	[0.580]	[0.607]
Age	1.002	0.822	0.875	0.927	1.083	0.914
	[0.116]	[0.120]	[0.104]	[0.110]	[0.106]	[0.111]
Female * age	1.026	1.158	1.396*	1.287$^{\dagger }$	1.050	1.067
	[0.162]	[0.164]	[0.144]	[0.151]	[0.150]	[0.157]
Residence	1.244	2.019*	1.237	1.101	1.043	1.205
	[0.291]	[0.297]	[0.269]	[0.273]	[0.272]	[0.278]
Prosthesis	0.738	0.830	1.255	0.848	0.843	1.074
	[0.336]	[0.334]	[0.294]	[0.303]	[0.319]	[0.309]
Time period	0.818	0.832	1.198	0.962	1.200	1.016
	[0.286]	[0.288]	[0.259]	[0.264]	[0.270]	[0.274]
N	223	223	204	202	218	211

Coefficients represent odds ratios; standard errors in squared brackets; Age numerically coded according to seven age groups; Residence equals one for patients living in Munich; Prosthesis equals one for hip replacement; Time period equals one if surgery was 12–17 months ago (compared to < 12 months). $$^{*}{{p}}<0.05; \,^{**}{{p}}<0.01; \,^{\dagger }{{p}}<0.1$$

The non-significant type of prosthesis dummies in all models were a first indication that hip and knee replacement patients did not perceive PROMs collection differently. This was confirmed when comparing the dichotomized survey responses based on Chi-square tests. No significant differences between types of replacement surgery emerged for any of the six survey questions.

## Discussion

Internationally, PROMs are increasingly being collected as a tool for assessing patient-relevant outcomes. While routine PROMs collection is established in other countries, the discussion about using PROMs in quality assurance in the German health care sector only just begun [3, 4]. In the present study, we examined experiences with and perception of PROMs collection in patients undergoing hip or knee replacement in Germany and provided evidence on the acceptability of PROMs in a clinical setting. Similar to previous findings [e.g., 10, 22, 20], our results indicated that patients generally experienced the PROMs collection as practicable and beneficial. However, around half of patients did not feel that PROMs collection had an impact on their involvement or their interaction with the physician.

We found no significant differences in survey responses between patients with hip or knee replacement. Positive evaluations of PROMs collection were also found in other disease areas such as rheumatology [11] or neurology [22].

The collection of a disease-specific and a generic quality of life instrument prior to replacement surgery was perceived to be practicable and of value to patients. This adds to the growing body of evidence of a generally positive patient experience of PROMs collection [10–12, 20, 22, 23]. Finding lower levels for the feeling of involvement and the perceived improved interaction with the physician should be interpreted in light of a previous study [10]. There, these levels were significantly higher in patients, who indicated that the PROM responses were reviewed by the physician. While further, especially qualitative research, seems warranted to examine this in more detail, this suggests that the explicit use of the collected PROMs by providers in their interaction with patients is to be recommended in a clinical setting. Concurrently, this has the potential to improve patient-provider communication and patient participation [8, 9].

A limitation of our study is the large lag of at least 6 months between PROM measurement and the administration of the survey about the PROMs collection. In other studies, both events took place on the same day [11]. Furthermore, the survey was administered online and only to patients who provided an e-mail address at their on-site visit, which likely led to a rather tech-savvy sample in this high-age population. Consequently, we might overestimate the acceptance of the digital PROMs collection. Further noteworthy limitations include the comparatively small sample size of our study and a rather low response rate. Lastly, no data were available on the actual usage of PROMs in the interaction with the physician.

To conclude, similar to the development in other countries, orthopedic procedures could serve as a starting point for broader use and routine PROMs collection in Germany. Lacking a national strategy, bottom-up and research initiatives or institutions like the IQTIG or Federal Joint Committee will need to advance the use and collection of PROMs in Germany [3]. This will become essential in the ongoing endeavour to further move towards patient-centered health care in Germany.

## Data Availability

The data and code for this analysis are available upon reasonable request.
